# Elevated homocysteine levels are associated with increased diabetes risk in patients with hypertension: a multicenter longitudinal study

**DOI:** 10.3389/fcvm.2026.1851360

**Published:** 2026-06-09

**Authors:** Ya He, Xianwei Tian, Chenru Zhao, Meng Chen, Hongyan Zhang

**Affiliations:** 1 Heart, Lung and Vessels Center, Sichuan Provincial People’s Hospital, University of Electronic Science and Technology of China, Chengdu, Sichuan, China; 2 Department of Cardiology, Xinxiang Central Hospital, Xinxiang, Henan, China; 3 Department of Pharmacy, Qingdao Central Hospital, University of Health and Rehabilitation Sciences, Qingdao, Shandong Province, China; 4 Department of Cardiology, Suzhou Hospital of Anhui Medical University, Suzhou, China

**Keywords:** cardiovascular disease, diabetes, homocysteine, hypertension, insulin resistance

## Abstract

**Background:**

Previous studies have consistently confirmed an association between homocysteine (Hcy) and hypertension. However, whether homocysteine is also associated with diabetes remains controversial. The aim of this study was to investigate the potential relationship between Hcy and the risk of diabetes in patients with hypertension.

**Methods:**

This study enrolled 5,981 hypertensive patients from three centers. Multivariable Cox regression was used to assess the association between Hcy and diabetes risk. Kaplan–Meier (KM) curves compared diabetes risk across groups, while restricted cubic spline (RCS) analysis with sex stratification explored threshold effects, followed by two-stage comparisons. Subgroup analyses based on Hcy status, baseline characteristics, disease status, and medication use evaluated result robustness.

**Results:**

Elevated Hcy was significantly associated with increased diabetes risk. Each 1 μmol/L and 1-standard deviation increase in Hcy corresponded to 3.3% and 27.7% higher risk, respectively. The highest diabetes risk during follow-up was observed in the group with elevated Hcy. RCS analysis revealed thresholds of 15.2 μmol/L in females and 15.9 μmol/L in males, beyond which diabetes risk increased markedly. Consistent findings across subgroup analyses further confirmed the robustness of the results.

**Conclusion:**

Elevated Hcy levels in patients with hypertension are significantly associated with an increased future risk of diabetes, with this association becoming particularly pronounced when Hcy levels exceed 15.2 μmol/L in females and 15.9 μmol/L in males. This finding further suggests that lowering Hcy levels may not only contribute to blood pressure control but also reduce the risk of diabetes, exerting a dual protective effect.

## 1 Introduction

Diabetes is one of the most prevalent and common chronic diseases, characterized by sustained hyperglycemia and clinically manifested by polydipsia, polyphagia, polyuria, and weight loss (1, 2). Poor long-term glycemic control and insulin resistance (IR) can lead to a range of complications, affecting not only the retina and peripheral nerves but also resulting in chronic kidney disease and even end-stage renal disease (3–5). In addition, IR may induce lipid metabolism disorders, which in turn cause vascular damage and blockage, ultimately increasing the risk of cardiovascular disease (CVD) (6, 7).

Hypertension, another highly prevalent chronic condition, often coexists with diabetes in middle-aged and older populations and can likewise lead to a series of target organ injuries (8–10). When the two diseases co-occur, they further exacerbate the risk of multiple complications, adverse outcomes, and mortality (8, 11, 12). With accelerating population aging, continuously increasing life expectancy, and rising living standards, the incidence of both diseases has been rising rapidly year by year (8, 13). Therefore, effectively preventing the onset of diabetes in patients with hypertension and delaying the progression of diabetes-related events in those with comorbid hypertension and diabetes hold significant clinical value and public health importance for improving life expectancy and quality of life in this population.

Previous perspectives attributed the occurrence of diabetes in patients with hypertension primarily to long-term high-sugar diets, obesity-induced IR and metabolic disorders, as well as family history related to diabetes (14–16). Although interventions targeting these factors—such as lifestyle modification, risk factor control, weight reduction, and metabolic regulation—can reduce risk to some extent, diabetes events still occur in a subset of patients. This suggests that the risk factors for diabetes in the hypertensive population may extend far beyond those traditionally recognized.

In recent years, expanding research perspectives have proposed that elevated levels of homocysteine (Hcy), a non-essential amino acid, may be associated with glucose metabolism disorders and IR, thereby influencing the development of diabetes (17–19). Clinical studies have also provided supporting evidence (20–22). A case–control study reported significantly higher diabetes prevalence among individuals with higher Hcy levels (20). Additionally, studies in pregnant women have shown that elevated Hcy increases the risk of gestational diabetes mellitus (21). Notably, Hcy has also been found to exacerbate vascular damage, renal function deterioration, and retinopathy progression in patients with diabetes, further underscoring its pathological significance (17, 23–25). Meanwhile, Hcy has been established as an important and independent risk factor for hypertension (26, 27). Given this context, investigating whether Hcy increases the risk of diabetes specifically in patients with hypertension holds significant research value and clinical relevance. Therefore, this study aims to explore the potential association between Hcy levels and the risk of future diabetes in patients with hypertension, with the goal of providing evidence to support the hypothesis that lowering Hcy may not only contribute to blood pressure (BP) control but also reduce the risk of incident diabetes.

## 2 Material and methods

### 2.1 Study population

**Figure 1:**
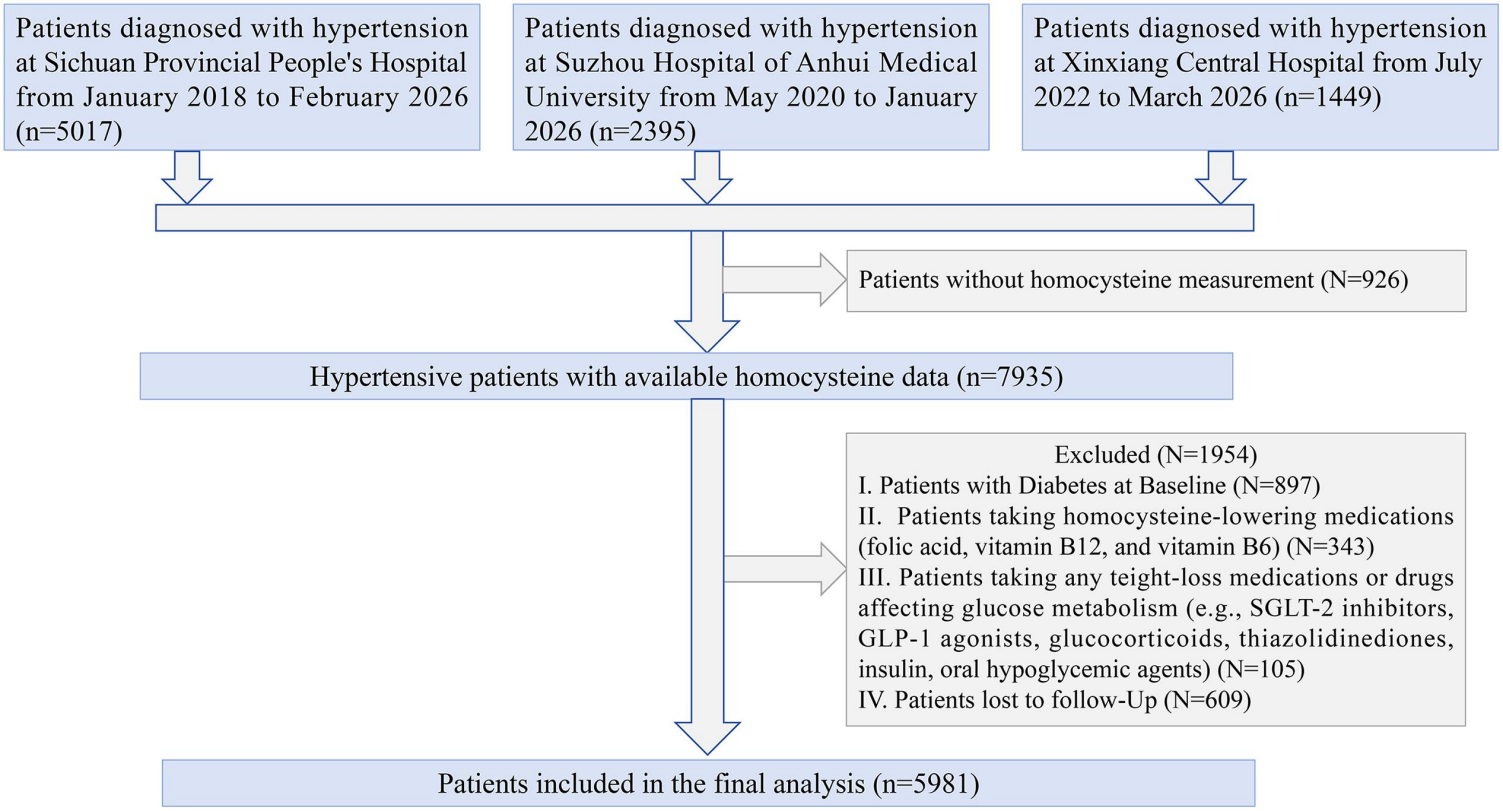
Patient selection flowchart.

A total of 8,861 patients with hypertension from three centers were initially enrolled in this study. First, 926 patients were excluded due to missing Hcy data, leaving 7,935 hypertensive patients with available Hcy measurements. Subsequently, we sequentially excluded patients with diabetes at baseline, those taking homocysteine-lowering medications, those receiving any weight-loss medications or drugs affecting glucose metabolism, and those lost to follow-up. After applying these rigorous exclusion criteria, a total of 5,981 patients with hypertension met the eligibility criteria for final analysis. Figure 1 presents the patient selection flowchart.

This study was conducted in strict accordance with the clinical research guidelines of the Declaration of Helsinki. The study protocol was approved by the ethics committees of three medical centers: Sichuan Provincial People's Hospital (approval no. CRC2018-0109), Suzhou Hospital of Anhui Medical University (approval no. SZ.N.202005), and Xinxiang Central Hospital (approval no. NXCH20220705).

### 2.2 Data collection

Patient information was collected through medical records from multiple centers, hospital electronic medical records, insurance claim data, as well as telephone and regular follow-up visits. The collected data primarily included general characteristics, baseline information, medical history, medication use, and laboratory findings.

General characteristics, including height, weight, body mass index (BMI), BP, and FPG, were measured by trained nurses following standardized protocols. Laboratory measurements comprised liver function, total cholesterol (TC), triglycerides (TG), high-density lipoprotein cholesterol (HDL-C), low-density lipoprotein cholesterol (LDL-C), hemoglobin A1c (HbA1c), and Hcy. Medical history focused on the prevalence of coronary heart disease (CHD) and hyperlipidemia. Medication information covered lipid-lowering drugs, antiplatelet agents, and various antihypertensive medications, including diuretics, beta-blockers, calcium channel blockers, and angiotensin-converting enzyme inhibitors (ACEIs)/angiotensin receptor blockers (ARBs).

### 2.3 Outcome

The outcome of this study was the occurrence of type 2 diabetes events during the entire follow-up period. The diagnosis of the endpoint event—diabetes—was strictly determined according to the diagnostic guidelines issued by the Endocrine Society, based on a combination of typical clinical symptoms, FPG, oral glucose tolerance test, and HbA1c levels (28, 29).

All patients were followed up from enrollment until the earliest occurrence of the following: the date of the last follow-up visit, the date of the first diagnosis of diabetes, or the end date of the overall study.

### 2.4 Statistical analysis

Patients with hypertension were divided into four equal groups based on the quartiles of Hcy levels, and baseline characteristics were compared across the four groups. The proportional hazards assumption for Cox regression was first tested, and the results confirmed that the assumption was satisfied. Therefore, multivariable stepwise-adjusted Cox regression analysis was employed to investigate the association between Hcy and the risk of incident diabetes. Additionally, Kaplan–Meier (KM) curves were plotted to compare the cumulative incidence of diabetes across different groups.

To further explore the dose-response relationship between Hcy and diabetes, restricted cubic spline (RCS) analysis was performed to validate the association, and the inflection points were further examined by sex stratification. Two-stage effect comparisons were conducted based on the identified inflection points. Finally, extensive subgroup analyses were performed according to different baseline characteristics and medication use to assess the robustness of the findings.

All statistical analyses were performed using R software (version 4.2.3). Two-sided *P* < 0.05 was considered statistically significant.

## 3 Results

### 3.1 Comparison of baseline characteristics of the population among the four groups

A total of 5,981 hypertensive patients from three centers were enrolled in this study. During a median follow-up of 3.8 years, 1,541 patients developed incident diabetes, with an incidence rate of 22.11 per 100 person-years. Table 1 presents the comparison of baseline characteristics across the four groups stratified by quartiles of Hcy levels.

**Table 1:** Baseline characteristics of the study participants.

Characteristic	Overall	Q1	Q2	Q3	Q4	*P* value
Number	5,981	1,495	1,495	1,495	1,496	
Sex (%)						<0.001
Female	2,721 (45.49%)	734 (49.10%)	703 (47.02%)	661 (44.21%)	623 (41.64%)	
Male	3,260 (54.51%)	761 (50.90%)	792 (52.98%)	834 (55.79%)	873 (58.36%)	
Age (years)	59.34 ± 8.27	59.75 ± 8.57	59.91 ± 8.58	58.61 ± 7.78	59.08 ± 8.07	<0.001
Current smoking (%)	1,225 (20.48%)	306 (20.47%)	263 (17.59%)	300 (20.07%)	356 (23.80%)	<0.001
Current drinking (%)	1,345 (22.49%)	318 (21.27%)	316 (21.14%)	336 (22.47%)	375 (25.07%)	0.036
BMI (kg/m^2^)	25.79 ± 4.00	25.98 ± 4.36	25.98 ± 3.97	25.69 ± 3.82	25.50 ± 3.80	0.001
SBP (mmHg)	145.25 ± 18.71	140.97 ± 18.30	143.70 ± 17.48	147.10 ± 18.60	149.23 ± 19.36	<0.001
DBP (mmHg)	88.00 ± 13.56	87.27 ± 12.89	87.30 ± 13.59	88.69 ± 13.67	88.74 ± 14.00	<0.001
Laboratory tests						
ALT (U/L)	17.00 (12.00−27.00)	16.00 (12.00−27.00)	16.00 (12.36–27.30)	17.00 (12.00–25.00)	17.00 (12.00–28.00)	0.878
AST (U/L)	18.00 (15.00–23.69)	17.19 (15.00–23.05)	18.00 (15.00–23.00)	18.00 (15.00–23.00)	18.70 (15.00–25.00)	0.014
TC (mmol/L)	4.11 ± 0.93	4.06 ± 0.87	4.08 ± 0.95	4.14 ± 0.98	4.15 ± 0.90	0.015
TG (mmol/L)	0.65 (0.55–1.43)	0.64 (0.55–1.30)	0.65 (0.55–1.48)	0.65 (0.54–1.58)	0.67 (0.57–1.42)	0.039
LDL-C (mg/dL)	2.72 ± 0.79	2.66 ± 0.81	2.70 ± 0.79	2.76 ± 0.74	2.78 ± 0.79	<0.001
HDL-C (mg/dL)	1.18 ± 0.28	1.18 ± 0.29	1.18 ± 0.29	1.19 ± 0.28	1.16 ± 0.27	0.009
FPG (mmol/L)	4.74 ± 0.89	4.65 ± 0.77	4.73 ± 0.89	4.75 ± 0.89	4.81 ± 1.00	<0.001
HbA1c (%)	6.30 ± 0.96	6.23 ± 0.93	6.23 ± 0.96	6.36 ± 0.94	6.40 ± 1.00	<0.001
Hcy (μmol/L)	15.60 (13.04–19.60)	11.50 (10.50–12.20)	14.11 (13.70–14.92)	17.20 (16.30–18.10)	25.36 (21.66–32.33)	<0.001
Medical history						
CHD (%)	1,860 (31.10%)	407 (27.22%)	428 (28.61%)	512 (34.25%)	513 (34.31%)	<0.001
Hyperlipidemia (%)	2,716 (45.41%)	648 (43.34%)	664 (44.41%)	685 (45.82%)	719 (48.06%)	<0.001
Medications						
Lipid-lowering drugs (%)	1,782 (29.79%)	375 (25.08%)	421 (28.16%)	480 (32.11%)	506 (33.82%)	<0.001
Antiplatelet drugs (%)	1,473 (24.63%)	315 (21.07%)	367 (24.55%)	389 (26.02%)	402 (26.87%)	<0.001
Diuretics (%)	970 (16.22%)	239 (15.99%)	219 (14.65%)	260 (17.39%)	252 (16.84%)	0.194
Beta-blockers (%)	1,552 (25.95%)	313 (20.94%)	346 (23.14%)	424 (28.36%)	469 (31.35%)	<0.001
Calcium channel blockers (%)	2,865 (47.90%)	690 (46.15%)	750 (50.17%)	713 (47.69%)	712 (47.59%)	0.173
ACEIs/ARBs (%)	2,838 (47.45%)	697 (46.62%)	702 (46.96%)	747 (49.97%)	692 (46.26%)	0.157

Data are presented as mean ± standard deviation, median (interquartile range), or as numbers, and percentages.

BMI, body mass index; SBP, systolic blood pressure; DBP, diastolic blood pressure; ALT, alanine transaminase; AST, aspartate transaminase; TC, total cholesterol; TG, triglyceride; LDL-C, low-density lipoprotein cholesterol; HDL-C, high-density lipoprotein cholesterol; FPG, fasting plasma glucose; HbA1c, hemoglobin A1c; Hcy, homocysteine; CHD, coronary heart disease; ACEIs, angiotensin-converting enzyme inhibitors; ARBs, angiotensin receptor blockers.

Compared with patients in the lower Hcy group, those in the higher Hcy group had a higher proportion of males, were younger in age, but exhibited higher rates of smoking and alcohol consumption, as well as higher BP levels. Regarding laboratory parameters, the higher Hcy group also showed relatively higher levels of liver enzymes, TC, TG, LDL-C, FPG, and HbA1c, with Hcy levels being significantly elevated. In terms of medical history, this group had a higher prevalence of CHD and hyperlipidemia. Consistently, the use of lipid-lowering drugs, antiplatelet agents, and beta-blockers was significantly higher in this group.

### 3.2 Impact of elevated Hcy on the risk of diabetes in patients with hypertension

**Figure 2:**
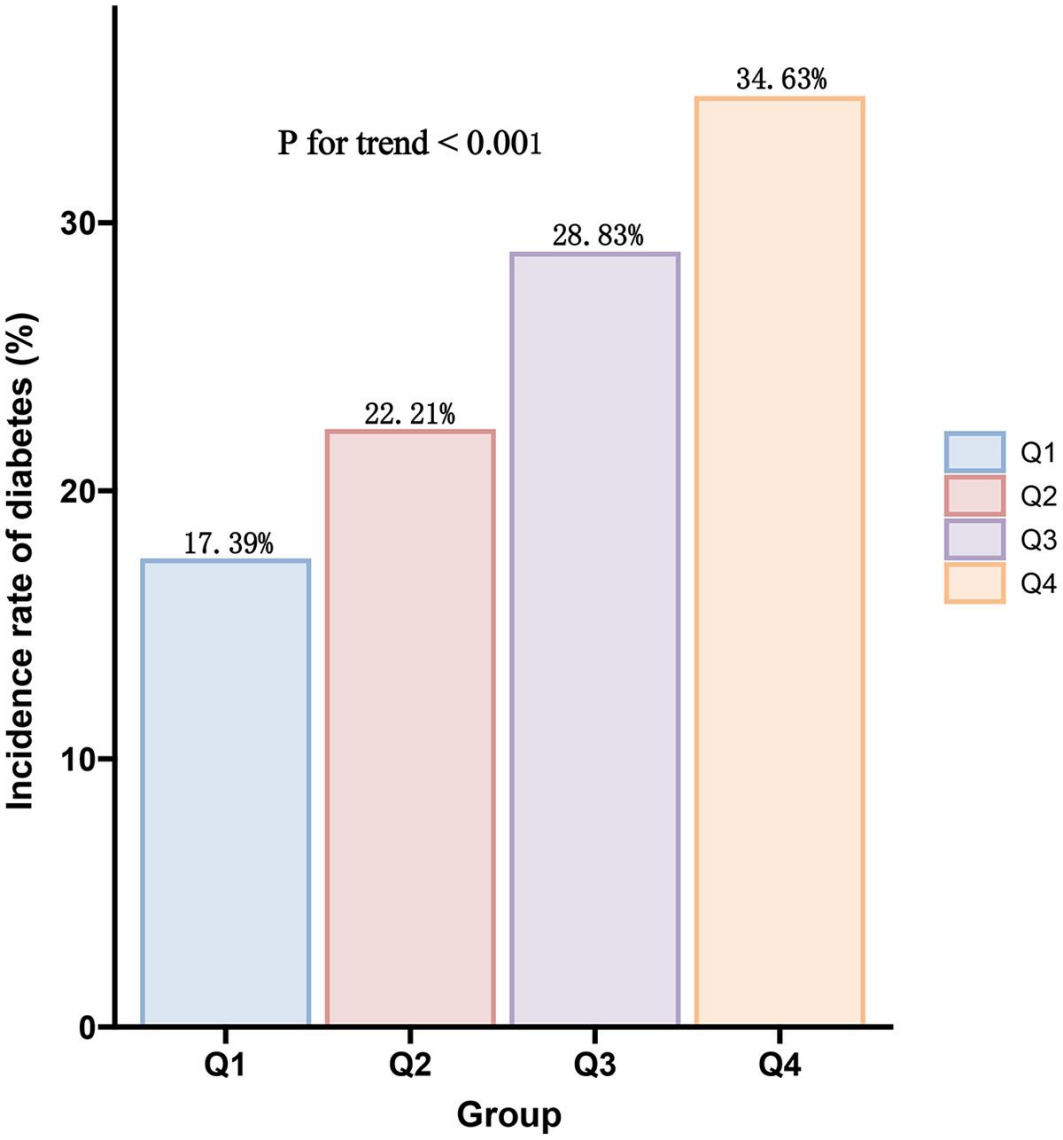
Diabetes incidence by quartile groups.

To investigate the association between Hcy levels and the risk of diabetes in patients with hypertension, we first compared the incidence of diabetes events across the different groups. The results showed a gradually increasing trend in diabetes incidence from the lowest quartile (Q1) to the highest quartile (Q4), with a clear dose-dependent pattern (Figure 2).

Based on this stepwise increasing trend in incidence across groups, we further employed multivariable stepwise-adjusted Cox regression analysis to explore the association. In the unadjusted Model 1, each 1 μmol/L increase in Hcy was associated with a 3.4% increase in diabetes risk [hazard ratio (HR) = 1.034; 95% confidence interval (CI): 1.035–1.049] (Table 2). In the fully adjusted Model 5, each 1 μmol/L increase in homocysteine remained associated with a 3.3% increase in risk (HR = 1.033; 95%CI: 1.028–1.039) (Table 2). Furthermore, each 5 μmol/L increase and each 1-standard deviation increase in Hcy were associated with 17.8% (HR = 1.178; 95%CI: 1.146–1.211) and 27.7% (HR = 1.277; 95%CI: 1.224–1.331) increases in risk, respectively (Table 2).

**Table 2:** Association between elevated Hcy and diabetes risk in hypertensive patients.

Diabetes	Model 1	Model 2	Model 3	Model 4	Model 5
	HR (95% CI) P	HR (95% CI) P	HR (95% CI) P	HR (95% CI) P	HR (95% CI) P
Hcy (per 1-μmol/L increase)	1.034 [1.028, 1.040] < 0.001	1.034 [1.028, 1.040] < 0.001	1.034 [1.028, 1.040] < 0.001	1.034 [1.028, 1.039] < 0.001	1.033 [1.028, 1.039] < 0.001
Hcy (per 5-μmol/L increase)	1.185 [1.152, 1.218] < 0.001	1.181 [1.149, 1.215] < 0.001	1.181 [1.148, 1.214] < 0.001	1.180 [1.148, 1.214] < 0.001	1.178 [1.146, 1.211] < 0.001
Hcy (per 1-SD increase)	1.287 [1.235, 1.342 < 0.001	1.282 [1.229, 1.337] < 0.001	1.281 [1.228, 1.335] < 0.001	1.280 [1.228, 1.334] < 0.001	1.277 [1.224, 1.331] < 0.001
Quartiles of Hcy					
Quartile 1	Reference	Reference	Reference	Reference	Reference
Quartile 2	1.473 [1.251, 1.733] < 0.001	1.448 [1.231, 1.705] < 0.001	1.421 [1.207, 1.674] < 0.001	1.421 [1.207, 1.672] < 0.001	1.411 [1.199, 1.662] < 0.001
Quartile 3	1.726 [1.478, 2.016] < 0.001	1.705 [1.460, 1.99] < 0.001	1.696 [1.452, 1.981] < 0.001	1.661 [1.423, 1.939] < 0.001	1.647 [1.412, 1.921] < 0.001
Quartile 4	2.215 [1.905, 2.574] < 0.001	2.194 [1.888, 2.549] < 0.001	2.190 [1.884, 2.545] < 0.001	2.185 [1.883, 2.537] < 0.001	2.163 [1.862, 2.513] < 0.001
P for trend	< 0.001	< 0.001	< 0.001	< 0.001	< 0.001

Model 1: no covariates were adjusted.

Model 2: age, sex, BMI, smoking status and drinking status were adjusted.

Model 3: Model 2 plus adjustment for SBP, DBP, TC, TG, LDL-C, HDL-C, FPG, and HbA1c.

Model 4: Model 3 plus adjustment for hyperlipidemia and CHD.

Model 5: Model 4 plus adjustment for use of antiplatelet drugs, lipid-lowering drugs, diuretics, beta-blockers, calcium channel blockers, and ACEIs/ARBs.

Hcy, homocysteine; HR, hazard ratio; CI, confidence interval.

**Figure 3:**
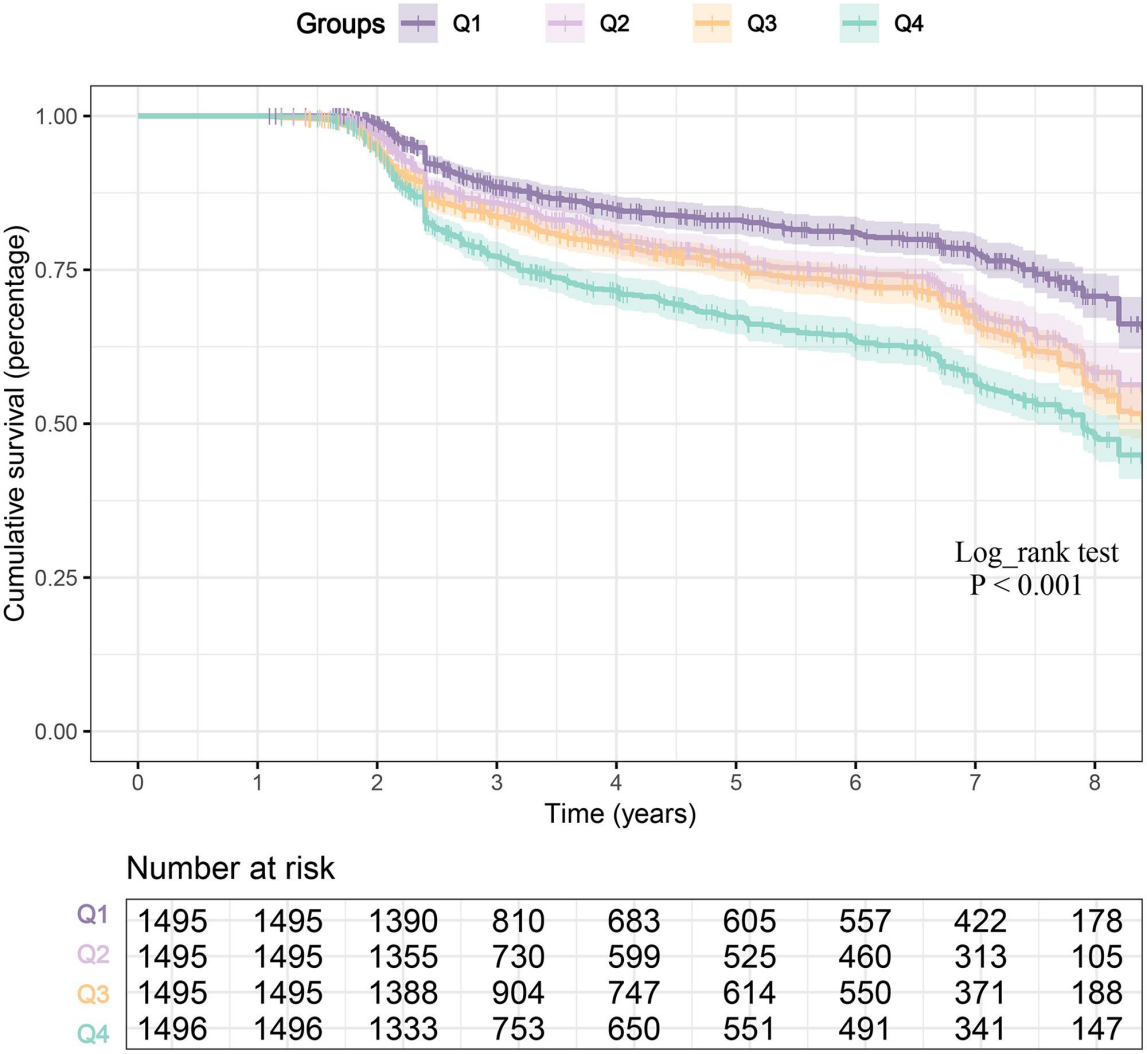
Kaplan–Meier curves for diabetes risk across the four groups.

When Hcy was analyzed as a categorical variable, compared with the lowest Q1 group, the Q2, Q3, and Q4 groups showed progressively increased risks of diabetes by 41.1% (HR = 1.441; 95%CI: 1.199–1.662), 64.7% (HR = 1.647; 95%CI: 1.412–1.921), and 116.3% (HR = 2.163; 95%CI: 1.862–2.513), respectively, with a significant dose-response trend (Table 2). To further validate this association, we plotted KM curves, which consistently demonstrated that the Q4 group had the highest risk of diabetes throughout the follow-up period (Figure 3).

### 3.3 Threshold effect of Hcy on diabetes risk

**Figure 4:**
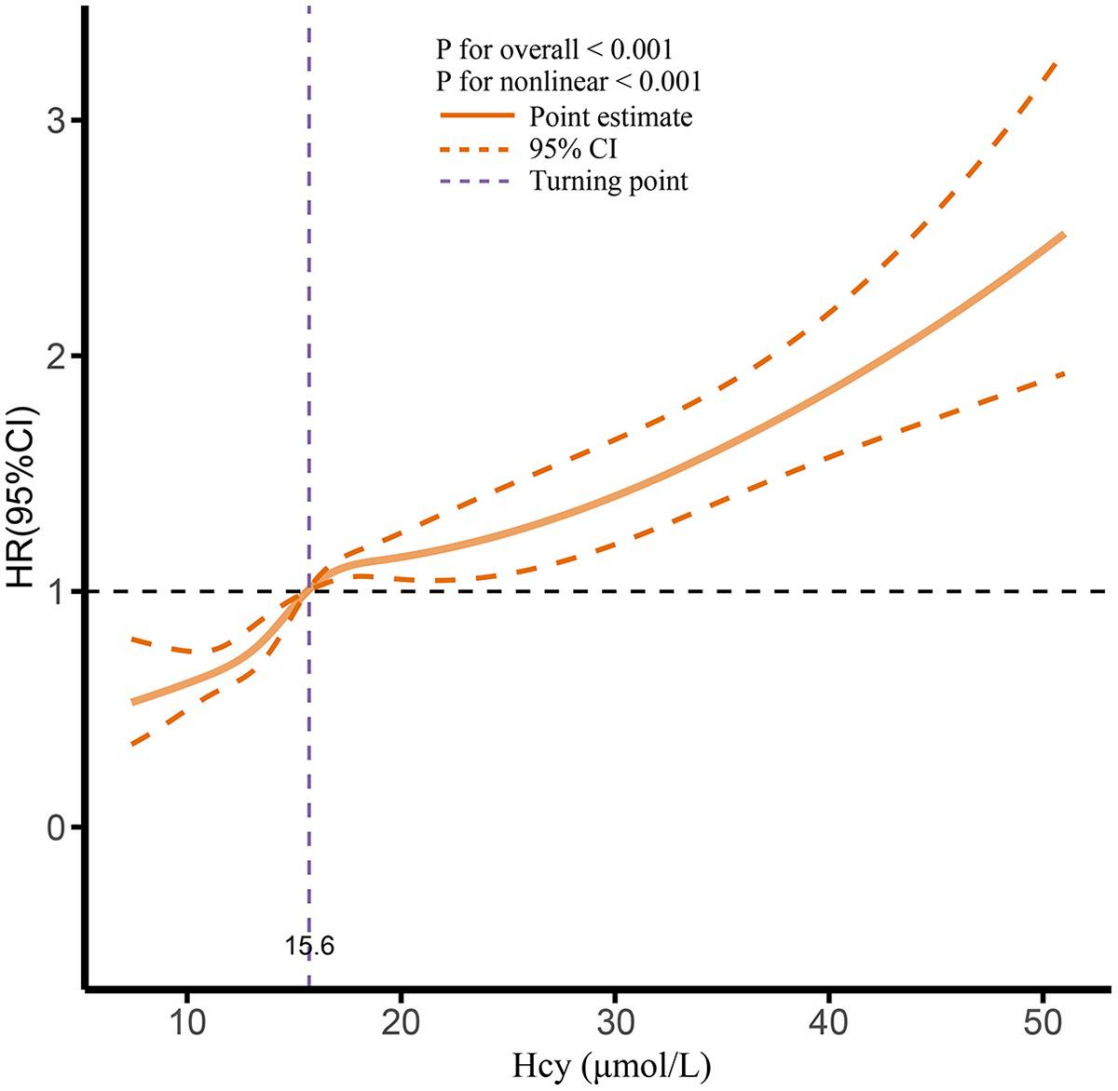
Dose-response and threshold effect of homocysteine on diabetes risk in the overall population.

**Figure 5:**
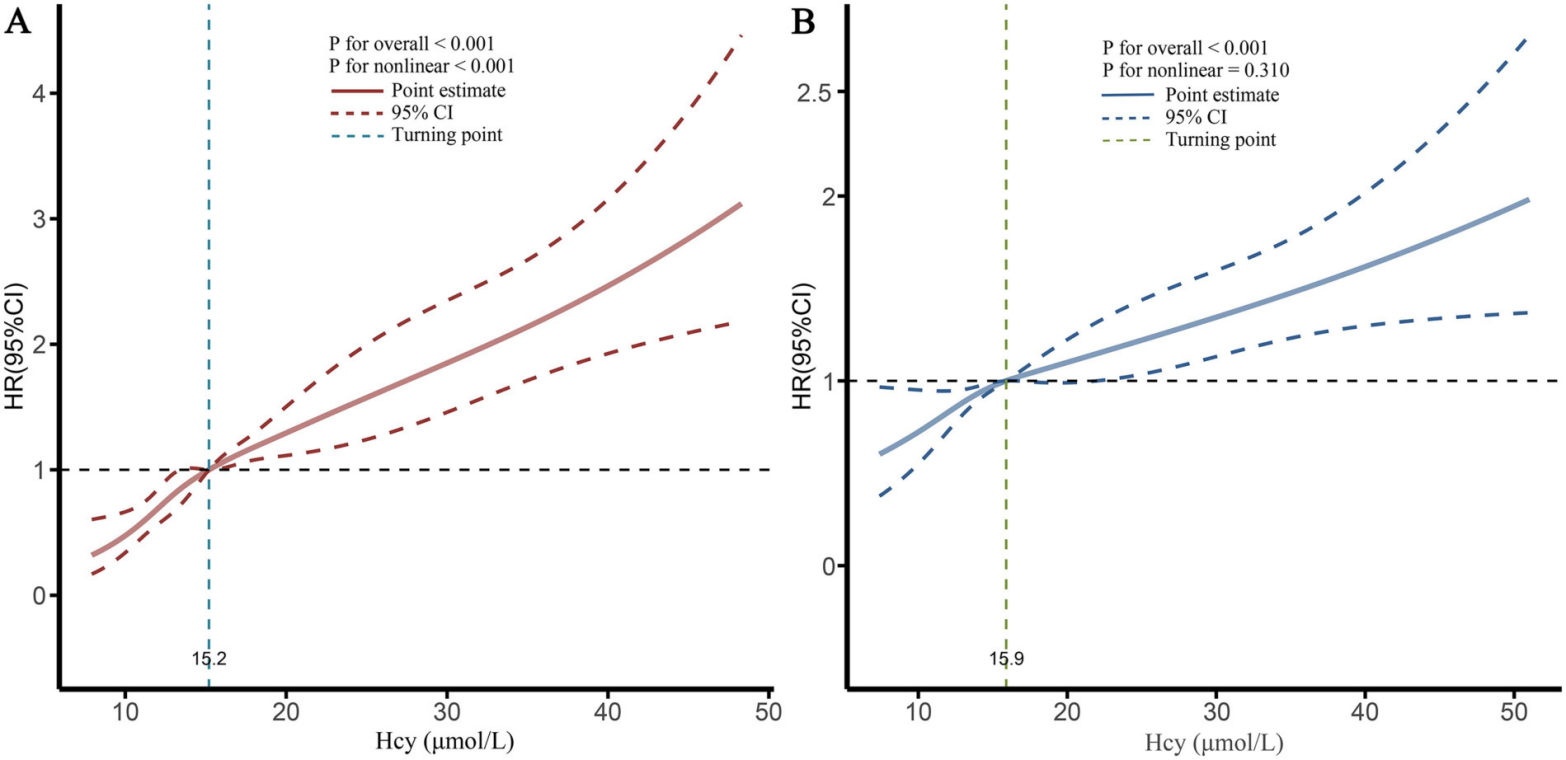
Sex-stratified analysis of dose-response and threshold effect of homocysteine on diabetes risk. **(A)**, Female; **(B)**, Male.

To further explore the dose-response relationship between Hcy and diabetes risk in patients with hypertension and to perform threshold analysis, we constructed RCS curves. In the overall population, a significant nonlinear association was observed between Hcy and diabetes risk, with a threshold of 15.6 μmol/L (Figure 4). Considering the potential influence of sex, we performed sex-stratified RCS analyses. The results showed a dose-response relationship between Hcy levels and diabetes risk in both sexes, with a threshold of 15.2 μmol/L in females and a relatively higher threshold of 15.9 μmol/L in males (Figure 5).

Based on the identified thresholds, we further conducted two-stage comparative analyses before and after the thresholds. In the overall population, individuals with Hcy levels above the threshold (15.6 μmol/L) had a 1.552-fold higher risk of diabetes compared with those below or equal to the threshold (Table 3). In sex-stratified analyses, females with Hcy levels above the threshold (15.2 μmol/L) had a 1.73-fold higher risk, while males with levels above the threshold (15.9 μmol/L) had a 1.278-fold higher risk (Table 3). These findings collectively indicate that in patients with hypertension, once Hcy levels exceed a certain threshold, the risk of diabetes increases significantly.

**Table 3:** Association between elevated Hcy and diabetes risk in hypertensive patients based on the turning point.

Diabetes	Model 1	Model 2	Model 3	Model 4	Model 5
	HR (95% CI) P	HR (95% CI) P	HR (95% CI) P	HR (95% CI) P	HR (95% CI) P
Overall					
Turning point	15.6	15.6	15.6	15.6	15.6
<= 15.6 (μmol/L)	Reference	Reference	Reference	Reference	Reference
>15.6 (μmol/L)	1.630 [1.470, 1.808] < 0.001	1.609 [1.451, 1.784] < 0.001	1.601 [1.444, 1.776] < 0.001	1.563 [1.409, 1.733] < 0.001	1.552 [1.401, 1.719] < 0.001
Female					
Turning point	15.2	15.2	15.2	15.2	15.2
<= 15.2 (μmol/L)	Reference	Reference	Reference	Reference	Reference
>15.2 (μmol/L)	1.940 [1.650, 2.280] < 0.001	1.905 [1.620, 2.239] < 0.001	1.778 [1.515, 2.086] < 0.001	1.748 [1.490, 2.051] < 0.001	1.730 [1.476, 2.027] < 0.001
Male					
Turning point	15.9	15.9	15.9	15.9	15.9
<= 15.9 (μmol/L)	Reference	Reference	Reference	Reference	Reference
>15.9 (μmol/L)	1.308 [1.144, 1.496] < 0.001	1.305 [1.141, 1.493] < 0.001	1.286 [1.124, 1.471] < 0.001	1.281 [1.121, 1.465] < 0.001	1.278 [1.119, 1.460] < 0.001

Model 1: no covariates were adjusted.

Model 2: age, sex, BMI, smoking status and drinking status were adjusted.

Model 3: Model 2 plus adjustment for SBP, DBP, TC, TG, LDL-C, HDL-C, FPG, and HbA1c.

Model 4: Model 3 plus adjustment for hyperlipidemia and CHD.

Model 5: Model 4 plus adjustment for use of antiplatelet drugs, lipid-lowering drugs, diuretics, beta-blockers, calcium channel blockers, and ACEIs/ARBs.

Hcy, homocysteine; HR, hazard ratio; CI, confidence interval.

### 3.4 Subgroup analysis

Considering that the association between Hcy and diabetes may vary across different subgroups, we conducted a series of subgroup analyses. First, using 15 μmol/L as the cutoff value based on the diagnostic criteria for Hcy, participants were divided into a normal group and a hyperhomocysteinemia group. The results showed that compared with the normal group, the hyperhomocysteinemia group had a significantly higher risk of diabetes, with an increased risk of 62.0% (Table 4).

**Table 4:** Association between Hcy and diabetes risk in hypertensive patients.

Diabetes	Model 1	Model 2	Model 3	Model 4	Model 5
	HR (95% CI) P	HR (95% CI) P	HR (95% CI) P	HR (95% CI) P	HR (95% CI) P
Normal group	Reference	Reference	Reference	Reference	Reference
Hyperhomocysteinemia Group	1.706 [1.533, 1.899] < 0.001	1.678 [1.508, 1.868] < 0.001	1.676 [1.506, 1.865] < 0.001	1.633 [1.468, 1.817] < 0.001	1.620 [1.457, 1.800] < 0.001

Age, sex, BMI, smoking status, drinking status, SBP, DBP, TC, TG, LDL-C, HDL-C, hyperlipidemia, CHD, antiplatelet drugs, lipid-lowering drugs, diuretics, beta-blockers, calcium channel blockers, and ACEIs/ARBs were adjusted.

CI, confidence interval; Hcy, homocysteine; HR, hazard ratio.

**Figure 6:**
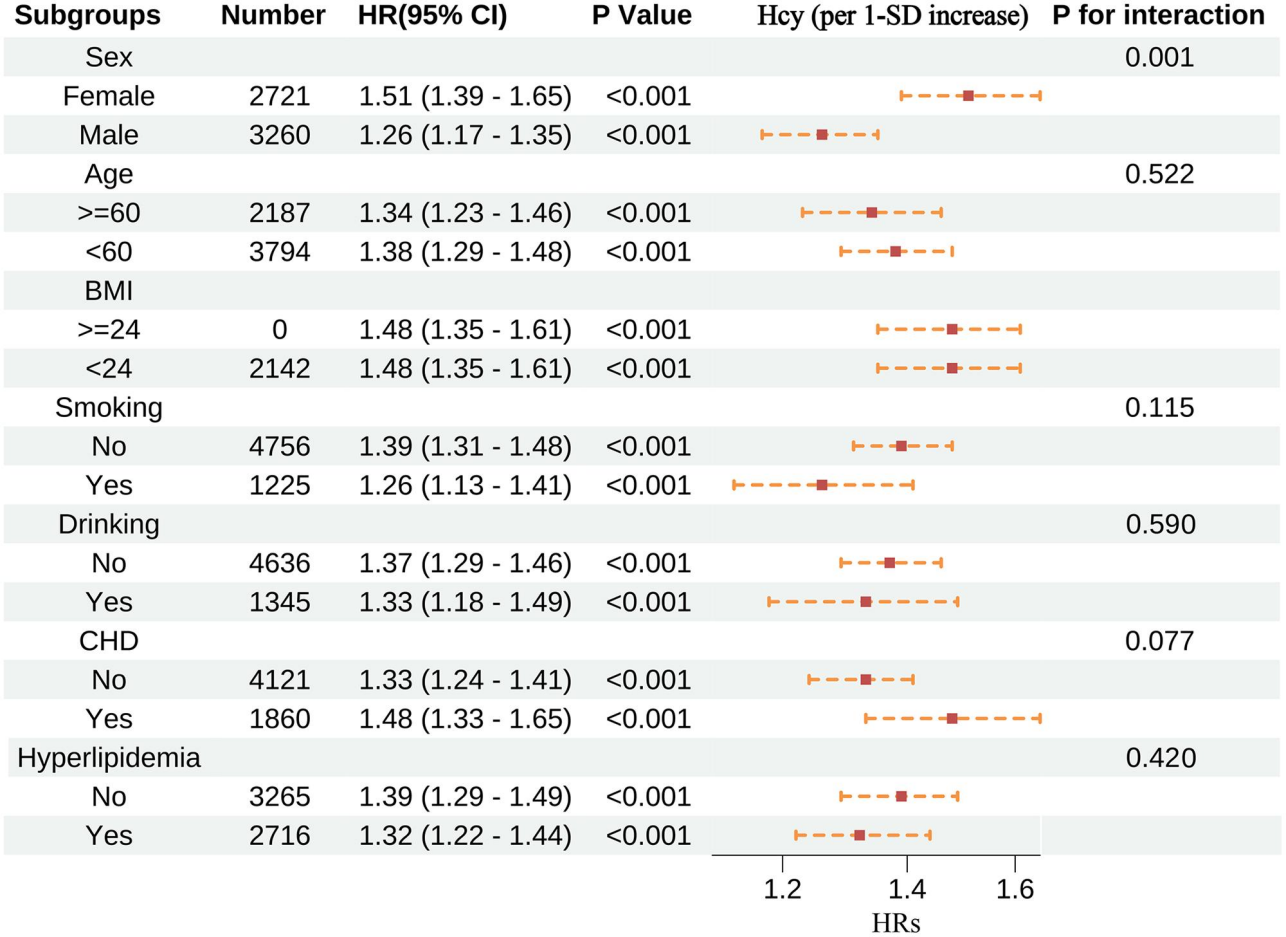
Subgroup analyses according to baseline characteristics and disease status.

**Figure 7:**
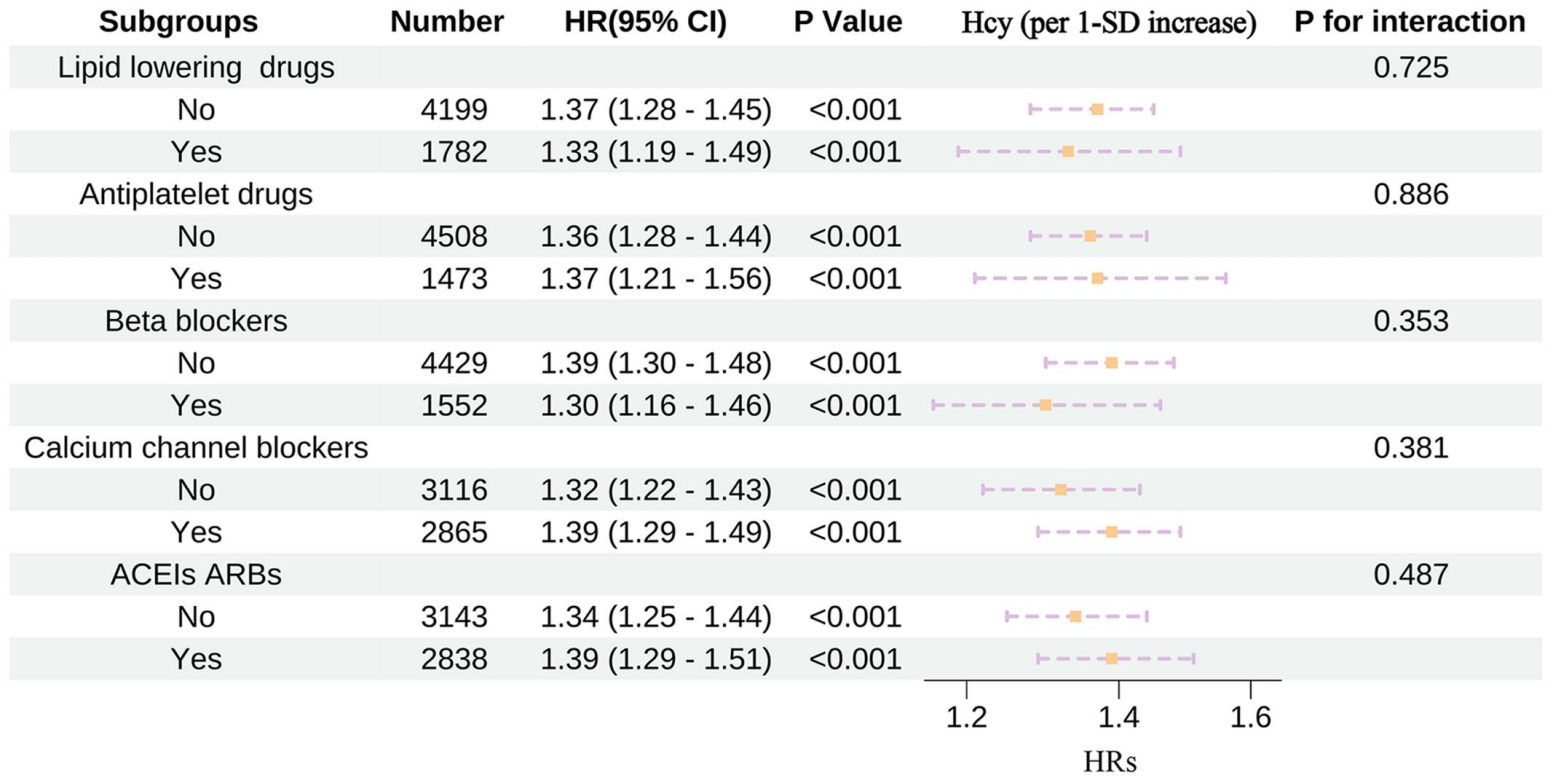
Subgroup analyses by medication use.

Given that variations in baseline characteristics and disease status among patients with hypertension may potentially confound our findings, we performed subgroup analyses stratified by sex, age, BMI, smoking status, alcohol consumption, as well as the presence of comorbid CHD and hyperlipidemia. The results across all subgroups were consistent with those observed in the overall analysis (Figure 6). Furthermore, as all participants in this study were patients with hypertension, the use of different medications represented an unavoidable confounding factor. We therefore conducted additional analyses stratified by medication use, and the results remained largely unchanged (Figure 7). These subgroup analyses further support the robustness of our findings, indicating that they were not substantially influenced by subgroup characteristics.

## 4 Discussion

Hcy, a non-essential amino acid in the human body, has been established as a risk factor for various CVDs, including hypertension (26, 30, 31). However, whether a significant association exists between Hcy and diabetes remains a topic of debate (20, 21, 32). Hypertension and diabetes often coexist in middle-aged and older populations, and the prevalence of this comorbidity continues to increase (33, 34). The concurrent presence of both conditions may further exacerbate adverse outcomes in patients with hypertension (10, 33, 35). Therefore, preventing the onset of diabetes in patients with hypertension holds considerable clinical significance. In this context, investigating the relationship between Hcy levels and diabetes in patients with hypertension is particularly warranted.

This study, based on a multicenter longitudinal cohort, aimed to explore the association between the two. The results demonstrated that elevated Hcy levels in patients with hypertension were strongly associated with an increased risk of subsequent diabetes, with each 1 μmol/L increase in homocysteine corresponding to a 3.3% increase in diabetes risk. Furthermore, a threshold effect was observed, with diabetes risk significantly increasing when Hcy levels exceeded 15.2 μmol/L in females and 15.9 μmol/L in males. These findings underscore the importance of maintaining Hcy at relatively low levels. Reducing homocysteine may not only help control BP and improve BP status but also potentially lower the future risk of diabetes, offering significant dual benefits.

In recent years, studies focusing on the relationship between Hcy and IR, as well as complications in diabetic populations, have gradually gained attention, providing new perspectives for further investigation into the association between Hcy and diabetes (18, 36–38). Evidence has shown a positive correlation between Hcy and IR, and mechanistic studies have revealed that Hcy may induce IR by inhibiting proinsulin receptor cleavage through protein S-homocysteinylation (18, 19, 36). Clinical studies have also yielded relevant findings (20–22). A case–control study conducted in western China reported a significantly higher prevalence of diabetes in the group with elevated Hcy levels, suggesting that Hcy may be an important risk factor for diabetes (20). Furthermore, studies in pregnant women have demonstrated that increased Hcy levels are associated with the development of gestational diabetes mellitus, highlighting the importance of monitoring and controlling Hcy levels during pregnancy (21, 22, 39, 40).

Notably, emerging evidence from studies on diabetic complications has shown that elevated Hcy levels in patients with diabetes are associated with a range of adverse outcomes, including poor glycemic control, vascular damage, exacerbation of retinopathy, and increased mortality risk (38, 41–43). Collectively, these findings further support a potential link between Hcy, IR, and diabetes, providing a strong rationale for conducting the present study and interpreting its findings.

The observed sex difference in the Hcy threshold for diabetes risk—15.2 μmol/L in females vs. 15.9 μmol/L in males—may be attributed to several biological factors. First, estrogen is known to modulate homocysteine metabolism by promoting its remethylation via the methionine synthase pathway, which may render women more sensitive to the deleterious effects of even modestly elevated Hcy levels once estrogenic protection is relatively diminished or overwhelmed (44, 45). Second, females generally have lower muscle mass and creatinine levels, leading to lower baseline Hcy concentrations; therefore, a relatively smaller absolute increase in Hcy may represent a greater proportional metabolic burden in women (46, 47). Third, sex differences in body fat distribution, insulin sensitivity, and inflammatory responses may contribute to a lower threshold for Hcy-associated diabetogenic effects in females (48, 49).

Elevated Hcy levels in patients with hypertension may promote the development of diabetes through multiple mechanisms. First, Hcy can inhibit proinsulin receptor cleavage via protein S-homocysteinylation, interfere with insulin signaling, and impair IRS-1 phosphorylation and PI3 K/Akt pathway activity, thereby inducing insulin resistance (50, 51). Second, Hcy induces oxidative stress and activates NF-κB-mediated inflammatory pathways, promoting the release of inflammatory cytokines, which in turn impair pancreatic β-cell function and induce apoptosis (52–54). Additionally, Hcy directly damages β-cell survival and insulin secretion through endoplasmic reticulum stress and mitochondrial dysfunction (53, 55, 56). Furthermore, Hcy induces endothelial dysfunction, reduces nitric oxide bioavailability, promotes vascular smooth muscle proliferation and atherosclerosis, indirectly affecting peripheral glucose uptake (41, 57). In the context of hypertension, Hcy acts synergistically with elevated blood pressure to exacerbate metabolic disturbances (26, 58). In summary, Hcy may contribute to the development and progression of diabetes in patients with hypertension through multiple pathways, including IR, oxidative stress and inflammation, β-cell injury, and vascular dysfunction.

The strength of this study lies in being the first to investigate the association between Hcy levels and the risk of diabetes in patients with hypertension—a population inherently prone to developing diabetes. This finding expands the etiological understanding of diabetes in hypertensive patients, suggesting that lowering Hcy levels may not only contribute to BP control but also reduce the risk of diabetes, which holds important clinical significance. Despite these strengths, several limitations should be acknowledged. First, only baseline Hcy levels were measured, precluding the assessment of dynamic changes during follow-up. Future studies could further explore the impact of Hcy trajectories on diabetes risk. Second, changes in dietary habits and physical activity patterns during follow-up may have influenced the results; however, such data were not available in this study, and future research should address this gap. Additionally, all participants were recruited from Chinese Han population, and whether the findings can be generalized to other populations or ethnic groups remains to be determined.

## 5 Conclusion

Elevated Hcy levels are strongly associated with an increased risk of diabetes in patients with hypertension, with a clear threshold effect. When Hcy levels exceed specific thresholds—15.2 μmol/L in females and 15.9 μmol/L in males—the increase in diabetes risk becomes particularly pronounced. These findings not only expand the scope of research on diabetes in patients with hypertension but also offer new insights for comprehensive management in this population. They suggest that lowering Hcy levels may confer multiple benefits: not only optimizing BP control but also potentially reducing the future risk of diabetes.

## Data Availability

The raw data supporting the conclusions of this article will be made available by the authors, without undue reservation.
